# The Role of Inflammation in Tinnitus: A Systematic Review and Meta-Analysis

**DOI:** 10.3390/jcm11041000

**Published:** 2022-02-14

**Authors:** Lilian M. Mennink, Marlien W. Aalbers, Pim van Dijk, J. Marc C. van Dijk

**Affiliations:** 1 Department of Neurosurgery, University Medical Center Groningen, University of Groningen, 9713 GZ Groningen, The Netherlands; m.w.aalbers@umcg.nl (M.W.A.); j.m.c.van.dijk@umcg.nl (J.M.C.v.D.); 2 Department of Otorhinolaryngology/Head & Neck Surgery, University Medical Center Groningen, University of Groningen, 9713 GZ Groningen, The Netherlands; p.van.dijk@umcg.nl; 3 Research School of Behavioral and Cognitive Neurosciences (BCN), University Medical Center Groningen, University of Groningen, 9713 AV Groningen, The Netherlands

**Keywords:** tinnitus, inflammation, microglia, astrocytes, cytokines, platelets

## Abstract

Subjective tinnitus is the perception of sound without the presence of an external source. Increasing evidence suggests that tinnitus is associated with inflammation. In this systematic review, the role of inflammation in subjective tinnitus was studied. Nine animal and twenty human studies reporting inflammatory markers in both humans and animals with tinnitus were included. It was established that TNF-α and IL-1β are increased in tinnitus, and that microglia and astrocytes are activated as well. Moreover, platelet activation may also play a role in tinnitus. In addition, we elaborate on mechanisms of inflammation in tinnitus, and discuss potential treatment options targeting inflammatory pathways.

## 1. Introduction

Subjective tinnitus is the perception of sound without the presence of an external source. Chronic tinnitus is prevalent among 5% to 42% of the general population, of which 3% to 30% report their tinnitus to be bothersome [1]. Quality of life is negatively affected by tinnitus in 1–4% of the general population, making tinnitus a substantial medical and socioeconomic problem [2]. Current treatments for tinnitus, such as hearing aids, cognitive behavioral therapy, and sound therapy aim to reduce tinnitus perception or to develop new coping strategies. However, there is no treatment that targets the pathophysiologic mechanism.

Knowledge of the pathophysiology of tinnitus is essential for the development of new treatment strategies. Previous research has established that tinnitus is associated with aberrant neuronal firing, regardless of the cause of tinnitus [3,4]. However, the processes that contribute to the emergence of aberrant neuronal firing in tinnitus are still a matter of debate.

The pathophysiology of tinnitus is closely related to that of acquired hearing loss, and there is increasing evidence that inflammation may contribute to the pathophysiology of hearing loss [5,6]. Accordingly, inflammation may also be one of the key processes in the development of tinnitus. Therefore, the aim of this review is to provide a systematic overview of the existing literature on inflammation in both human and experimental subjective tinnitus. Additionally, we elaborate on mechanisms of inflammation in tinnitus and discusses potential anti-inflammatory treatments for tinnitus.

## 2. Materials and Methods

### 2.1. Search Strategy

A systematic review on the role of inflammation in tinnitus was performed, with its methodology based on the framework of the Cochrane and PRISMA guidelines [7,8]. The search strategy (Box 1) was constructed with the assistance of a medical information specialist. The search was conducted in the PubMed and EMBASE databases and included articles published up to October 2021.

Box 1 **Search terms.**

**PubMed**

(“Tinnitus”[Mesh] OR tinnitus*[tiab])

AND

(neutrophil*[tiab] OR lymphocyt*[tiab] OR platelet*[tiab] OR cytokin*[tiab] OR interleukin*[tiab] OR ((tumor[tiab] OR tumour[tiab]) AND necrosis[tiab] AND factor*[tiab]) OR TNF[tiab] OR interferon*[tiab] OR chemokine*[tiab] OR COX [tiab] OR antibod*[tiab] OR microgli*[tiab] OR astrocyt*[tiab])

**EMBASE**

(‘tinnitus’/exp OR tinnitus*:ab,ti)

AND

(neutrophil*:ab,ti OR lymphocyt*:ab,ti OR platelet*:ab,ti OR cytokin*:ab,ti OR interleukin*:ab,ti OR ((tumor:ab,ti OR tumour:ab,ti) AND necrosis:ab,ti AND factor*:ab,ti) OR tnf:ab,ti OR interferon*:ab,ti OR chemokine*:ab,ti OR cox:ab,ti OR antibod*:ab,ti OR microgli*:ab,ti OR astrocyt*:ab,ti)

Cross-sectional, cohort, or case-control studies assessing inflammatory markers in subjective tinnitus in humans or animals were considered for the review, as well as interventional studies if they contained baseline measurements. Studies without accessible full text were excluded, as were studies lacking numerical data, addressing objective or pulsating tinnitus, tinnitus as a part of another disease (e.g., Meniere’s disease), or as side effect of a treatment, as well as papers in language other than English, Dutch, German, French or Italian.

### 2.2. Study Selection and Data Extraction

Two reviewers (LMM and MWA) screened titles and abstracts after removal of duplicates and thereafter independently evaluated the full-texts of potentially relevant articles. Disagreements were resolved by a discussion between the reviewers to reach a consensus. The reference lists of included articles were manually searched to identify potentially missing articles. We constructed a data extraction file to systematically extract data regarding study characteristics. Data extraction was done independently by the two reviewers.

### 2.3. Meta-Analysis

Due to large heterogeneity of the data, a meta-analysis could only be performed for the human complete blood count markers. The estimated effect size of mean platelet volume (MPV), platelet distribution width (PDW), platelet count and neutrophil-to-lymphocyte ratio (NLR) on tinnitus presence was determined using the mean difference (MD). As considerable between-study heterogeneity was anticipated, a random-effects model was used to pool effect sizes. The restricted maximum likelihood estimator was used to calculate the heterogeneity variance 𝜏^2^ [9]. Knapp-Hartung adjustments were used to calculate the confidence interval around the pooled effect [10]. Between-study heterogeneity variance was calculated with 𝜏^2^, and the proportion of total variability due to heterogeneity with *I*^2^. Prediction intervals around the pooled effect, which provide a range into which we can expect the effects of future studies to fall, were calculated as well. Next, the cause of heterogeneity was assessed. There are multiple options to analyze heterogeneity, and the choice for one option over another is arbitrary. Therefore, a multiverse analysis is performed to determine how sensitive the conclusions are to decisions on the statistical method to assess heterogeneity [11]. The performed methods in the multiverse analysis were outlier removal and influential case detection. The multiverse analysis gives an indication of the robustness of the conclusions. All statistical analyses were performed in R (v4.0.1) software, with the R-packages {meta} [12], {metafor} [13], and {dmetar} [14]. Statistical significance was established at *p* < 0.05.

Currently, no method of providing acceptable results for publication bias analyses exists when between-study heterogeneity is high (*I*^2^ > 75%) [15]. Therefore, no publication bias analysis has been performed.

## 3. Results

**Figure 1:**
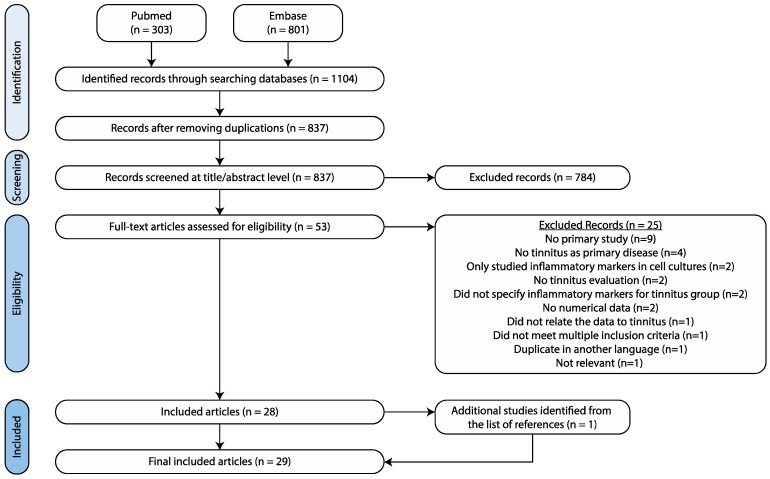
Search strategy.

The search yielded 303 hits in PubMed, as well as 801 hits in EMBASE (Figure 1). Duplicates were removed, resulting in 837 articles to be screened. During the initial title and abstract screening, 784 papers were excluded because they were out of scope. Of the remaining 53 full-text papers, 28 papers were included. The search within reference lists of the included papers resulted in one additional paper. As such, a total of 29 studies was included.

### 3.1. Animal Studies

#### 3.1.1. Study Characteristics

Nine experimental animal studies were included. The typical design of these studies is to induce tinnitus by salicylate, and to compare inflammatory markers between salicylate-treated animals and a control group. There was only one study in which traumatic loud noise was used to induce tinnitus [16]. A complete overview of all study characteristics is listed in Table 1.

#### 3.1.2. Salicylate-Induced Tinnitus

##### Study Characteristics

In the studies using the salicylate-induced tinnitus model, both behavioral evidence of tinnitus and changes in inflammatory markers almost always disappeared after a washout period. Salicylate did not induce behavioral signs of tinnitus in all animals. Those animals that failed to develop tinnitus did not show any differences in (gene) expression of inflammatory markers compared to healthy controls.

##### Cytokine Levels

(Gene) expression levels of Tumor Necrosis Factor-α (TNF-α) were increased in the cochlea and the inferior colliculus (IC) after four days of salicylate injections, in the auditory cortex (AC) after seven days of salicylate injections, and in the cochlear nucleus (CN) after seven and fourteen days of salicylate injections [17,18,19,20]. Acute treatment with a single injection of salicylate was associated with increased Interleukin (IL) 1β (gene) expression in the primary AC and medial geniculate body (MGB). Chronic injections for four days resulted in increased IL-1β gene expression in the cochlea and IC. However, seven days of injections did not change the expression in the MGB or primary AC [19,20,21]. Finally, TNF-α and IL-1β gene expression in the IC and cochlea were both positively associated with tinnitus scores after four days of salicylate injections [20]. In contrast, interferon-γ (IFN-γ) (gene) expression was decreased in the AC after seven days of salicylate injections. IL-6 expression showed no changes in the CN after salicylate injections [17].

##### Neuroglial Markers

In het MGB, a single injection of salicylate resulted in an increase in both the number of ionized calcium-binding adaptor protein-1 (Iba-1)-immunoreactive microglia and the expression level of Iba-1 up to 24 h after injection, and microglia showed morphological changes up to 4 h after injection. In the primary AC, an increase in both the number of Iba-1-immunoreactive microglia and the expression level of Iba-1 was present up to 24 h after injection. However, morphologically the microglia did not change. After seven days of salicylate administration, in the MGB the number of Iba-1-immunoreactive microglia and the expression level of Iba-1 were increased as well, and the microglia showed morphological changes. In the primary AC, the number of Iba-1-immunoreactive microglia and the expression level of Iba-1 were also increased, but morphologically the microglia did not change [21]. In contrast, Fang et al. (2016) showed a decrease in both the number of Iba-1-immunoreactive microglia and the expression level of Iba-1 after four and eight days of salicylate injections in the ventral CN [22].

**Table 1:** Animal studies assessing inflammatory markers in tinnitus. Results relating to animals with behavioral signs of tinnitus are marked by a light-grey background.

	Study	Animals	n	Tinnitus Induction	Measurement Timing	Tinnitus Evaluation	Tinnitus Present	Controls	Region of Interest	Method	Results	
											Significant	Not Significant
Salicylate induced tinnitus	Chen and Zheng, 2017 [18]	R	6	S 1d	+2 h	GPIAS	No	NM (n = 10)	AC	PCR		TNF-α, IL-6, IFN-γ
										Western blot		TNF-α, IL-6, IFN-γ
			10	S 7d	+2 h		Yes			PCR	TNF-α ↑, IFN-γ ↓	IL-6
										Western blot	TNF-α ↑, IFN-γ ↓	IL-6
			10	S 7d	+14 d		No			PCR		TNF-α, IL-6, IFN-γ
										Western blot		TNF-α, IL-6, IFN-γ
	Fang et al., 2016 [22]	R	10	S 4d	+4 h	GPIAS	Yes	Saline treated (n = 10)	Ventral CN	Western blot	Iba-1 ↓, GFAP ↑	
										IHC	Iba-1 ↓, GFAP ↑	
			10	S 8d	+4 h		Yes			Western blot	Iba-1 ↓, GFAP ↑	
										IHC	Iba-1 ↓, GFAP ↑	
			12	S 8d	+7 d		No			Western blot		Iba-1, GFAP
										IHC		Iba-1, GFAP
	Hu et al., 2014 [17]	R	6	S 1d	+1 d	GPIAS	No	No treatment (n = 10)	CN	PCR		TNF-α, IL-6
										Western blot		TNF-α, IL-6
			10	S 7d	+1 d		Yes			PCR	TNF-α ↑	IL-6
										Western blot	TNF-α ↑	IL-6
			10	S 14d	+1 d		Yes			PCR	TNF-α ↑	IL-6
										Western blot	TNF-α ↑	IL-6
										IHC	TNF-α ↑	IL-6
			6	S 14d	+15 d		No			PCR		TNF-α, IL-6
										Western blot		TNF-α, IL-6
			6	S 14d	+29 d		No			PCR		TNF-α, IL-6
										Western blot		TNF-α, IL-6
	Hwang et al., 2011a [20]	M	24	S 4d	+4 d	Behavioral conditioning	Yes	Saline treated (n = 24)	Cochlea	PCR	TNF-α ↑, IL-1β ↑ Tinnitus scores ↔ TNF-α Tinnitus scores ↔ IL-1β	
									IC	PCR	TNF-α ↑, IL-1β ↑ Tinnitus scores ↔ TNF-α Tinnitus scores ↔ IL-1β	
Salicylate induced tinnitus	Hwang et al., 2011b [23]	M	24	S 4d	+4 d	Behavioral conditioning	Yes	Saline treated (n = 24)	Cochlea	PCR		COX-2
									IC	PCR		COX-2
	Hwang et al., 2013 [19]	M	24	S 4d	+2 h	Behavioral conditioning	Yes	Saline treated (n = 24)	Cochlea	PCR	TNF-α ↑, IL-1β ↑	COX-2
									IC	PCR	TNF-α ↑, IL-1β ↑	COX-2
										Western blot		TNF-α, IL-1β, COX-2
	Hwang et al., 2017 [24]	R	15	S 3d	+0 d	Behavioral conditioning	Yes		Cochlea	PCR	TNF receptor-1 1.03, TNF receptor-2 66.86	
	Xia et al., 2020 [21]	R	4	S 1d	+2 h	GPIAS + PPI	Yes	Saline treated (n = 4)	AC1	PCR	IL-1β ↑	
										Western blot		GFAP, Iba-1
										IHC	astrocyte EP/C ↑, astrocyte LE/C ↑	GFAP, Iba-1, microglia EP/C, microglia LE/C, IL-1β
									MGB	PCR		IL-1β
										Western blot		GFAP, Iba-1
										IHC	microglia EP/C ↑, microglia LE/C ↑	GFAP, Iba-1, astrocyte EP/C, astrocyte LE/C, IL-1β
			4	S 1d	+4 h		Yes		AC1	PCR	IL-1β ↑	
										Western blot	GFAP ↑, Iba-1 ↑	
										IHC	GFAP ↑, Iba-1 ↑, astrocyte EP/C ↑, astrocyte LE/C ↑, IL-1β ↑	microglia EP/C, microglia LE/C
									MGB	PCR		IL-1β
										Western blot	Iba-1 ↑	GFAP
										IHC	GFAP ↑, Iba-1 ↑, microglia EP/C ↑, IL-1β ↑	astrocyte EP/C, astrocyte LE/C, microglia LE/C
Salicylate induced tinnitus	Xia et al., 2020 [21]		4	S 1d	+8 h		Yes		AC1	PCR		IL-1β
										Western blot	Iba-1 ↑	GFAP
										IHC	Iba-1 ↑, IL-1β ↑, astrocyte EP/C ↑	GFAP, astrocyte LE/C, microglia EP/C, microglia LE/C
									MGB	PCR	IL-1β ↑	
										Western blot	Iba-1 ↑	GFAP
										IHC	Iba-1 ↑	GFAP, IL-1β astrocyte EP/C, astrocyte LE/C, microglia EP/C, microglia LE/C
			4	S 1d	+24 h		No		AC1	PCR		IL-1β
										Western blot	Iba-1 ↑	GFAP
										IHC	Iba-1 ↑, IL-1β ↑	GFAP, astrocyte EP/C, astrocyte LE/C, microglia EP/C, microglia LE/C
									MGB	PCR	IL-1β ↑	
										Western blot	Iba-1 ↑	GFAP
										IHC	Iba-1 ↑	GFAP, IL-1β astrocyte EP/C, astrocyte LE/C, microglia EP/C, microglia LE/C
			4	S 7d	+0 d		Yes		AC1	PCR		IL-1β
										Western blot	GFAP ↑, Iba-1 ↑	
										IHC	GFAP ↑, Iba-1 ↑, astrocyte EP/C ↑, IL-1β ↑	astrocyte LE/C, microglia EP/C, microglia LE/C
									MGB	PCR		IL-1β
										Western blot	Iba-1 ↑	GFAP
										IHC	Iba-1 ↑, microglia EP/C ↑	GFAP, IL-1β astrocyte EP/C, astrocyte LE/C, microglia LE/C
Salicylate induced tinnitus	Xia et al., 2020 [21]		4	S 7d	+7 d		No		AC1	PCR		IL-1β
										Western blot		GFAP, Iba-1
										IHC		Iba-1, GFAP, IL-1β astrocyte EP/C, astrocyte LE/C, microglia EP/C, microglia LE/C
									MGB	PCR		IL-1β
										Western blot		GFAP, Iba-1
										IHC		Iba-1, GFAP, IL-1β astrocyte EP/C, astrocyte LE/C, microglia EP/C, microglia LE/C
Noise-induced tinnitus	Wang et al., 2019 [16]	M	4	2-h 8 kHz 112–114 dB unilateral sound (AS)	+12 h	GPIAS + PPI	?	NM (n = 4)	Bilateral AC1	PCR	TNF-α ↑ (ipsi > contra), NLRP3 ↑ (bilat.),	IL-1β, IL-18, TNF-α protein
			4		+1 d		?			PCR	TNF-α ↑ (bilat.), TNF-α protein ↑ (contra > ipsi)	IL-1β, IL-18, NLPR3
										IHC		Soma-to-whole cell size ratio
			4		+3 d		?			PCR	TNF-α protein ↑	TNF-α, IL-1β, IL-18, NLPR3
										IHC		Soma-to-whole cell size ratio
			4		+5 d		?			IHC	Soma-to-whole cell size ratio ↑ (contra > ipsi)	
			4		+10 d		Yes			PCR	TNF-α ↑ (bilat.), IL-1β ↑ (bilat.), IL-18 ↑ (bilat.), NLRP3 ↑ (bilat.), TNF-α protein ↑ (contra > ipsi)	

Abbreviations: AC, auditory cortex; AC1, primary auditory cortex; AS, left ear; CN, cochlear nucleus; COX, cyclooxygenase; d, days; EP/C, endpoints/cell; GFAP, glial fibrillary acidic protein; GPIAS, Gap-Prepulse Inhibition of the Acoustic Startle Reflex; h, hours; Iba, ionized calcium-binding adaptor; IC, inferior colliculus; IFN, interferon; IHC, immunohistochemistry; IL, interleukin; LE/C, process length/cell; M, mice; MGB, medial geniculate body; NM, treatment not mentioned; PCR, polymerase chain reaction; PPI, pre-pulse inhibition; R, rats; S xd, Salicylate treatment for x days; TNF, tumor necrosis factor; ↑, increased; ↓, decreased; ↔, positive correlation.

After a single injection of salicylate, in the MGB the number of glial fibrillary acidic protein (GFAP)-immunoreactive astrocytes was increased after four hours, but no difference was present in the expression level of GFAP. Moreover, the astrocytes showed no morphological changes. In the primary AC both the number of GFAP-immunoreactive astrocytes and the expression level of GFAP were increased after four hours. Additionally, the astrocytes showed morphological changes up to eight hours after injection. After seven days of salicylate administration, in the primary AC, both the number of GFAP-immunoreactive astrocytes and the expression level of GFAP were increased in the primary AC, and the astrocytes showed morphological changes. In the MGB, no differences were present in the number of GFAP-immunoreactive astrocytes and the expression level of GFAP, and astrocytes did not show morphological changes [21]. Moreover, in the ventral CN the GFAP-immunoreactive astrocytes were increased in number and showed stronger immunoreactivity after four and eight days of salicylate administration. The expression level of GFAP was increased as well [22].

##### Other Markers of Inflammation

In the cochlea and IC, no difference was present in the (gene) expression of cyclooxygenase-2 (COX-2), an enzyme that catalyzes the formation of prostaglandins [19,23].

#### 3.1.3. Noise-Induced Tinnitus

Only one study studied noise-induced tinnitus and performed a tinnitus evaluation [16]. Mice with tinnitus showed an increase in TNF-α gene expression in both auditory cortices twelve hours, one day and ten days after noise exposure, with a significantly higher expression in the ipsilateral AC than in the contralateral AC after twelve hours. TNF-α protein expression was increased after three and ten days. The NLPR3 inflammasome was increased after twelve hours and ten days, and IL-18 gene expression was increased after ten days. Five days after noise exposure, the microglial soma-to-whole cell size ratio was increased, indicating microglial activation. This was more pronounced on the contralateral side than the ipsilateral side.

### 3.2. Human Studies

#### 3.2.1. Study Characteristics

Six retrospective and fourteen prospective studies were included (Table 2). Twelve prospective studies were cross-sectional, comparing participants with and without tinnitus, and two prospective studies were non-randomized trials. From the non-randomized trials the baseline measurements were included. Sample sizes ranged from 26 to 287 subjects with tinnitus. Almost all studies excluded patients with known inflammatory diseases, except for Szczepek et al. (2014) and Bayraktar and Taşolar (2017) [25,26]. Audiometry was performed in almost all studies, but the hearing thresholds were not always reported. Both subjects with and without hearing loss were included, but no study matched subjects based on hearing loss. Bayraktar and Taşolar (2017), Kemal et al. (2016), Sarikaya et al. (2016), Savastano et al. (2006) and Savastano et al. (2007) included only normal hearing patients [25,27,28,29,30].

#### 3.2.2. Cytokine Levels

Two studies comparing cytokine levels between patients and healthy controls showed conflicting results [31,32]. Weber et al. (2002) showed significantly increased IL-6 blood levels, whereas Haider et al. (2020) did not show any difference [31,32]. IL-10 levels were equal according to Weber et al. (2002), but decreased in the study by Haider et al. (2020) [31,32]. Moreover, the latter demonstrated that the longer tinnitus existed, the higher IL-10 levels were. No differences were present in IL-1α, IL-1β, IL-2, IFN-γ, TNF-α and transforming growth factor-β (TGF-β) [31,32].

**Table 2:** Human studies assessing inflammatory markers in tinnitus. * Reported in mean ± SD unless otherwise stated.

Study	Participants					Controls		Tissue of Interest	Method	Results	
	n (M/F)	Age *	Tinnitus Location (uni/bi)	Tinnitus Duration	Tinnitus Score	n (m/f)	Age *			Significant	Not Significant
Avci, 2020 [33]	91 (34/57)	48.03 ± 15.12 y	67/24	7.17 ± 10.52 m	THI: 37.29 ± 15.22	65 (24/41)	47.55 ± 17.49 y	Blood	CBC	MPV ↑	neutrophils, lymphocytes, NLR, PDW, platelet count, PLR, RCDW, WBC
Bayraktar and Taşolar, 2017 [25]	40 (15/25)	41.00 ± 9.02 y	31/9	≥3 m	THI: slight (n = 10), mild (n = 11), moderate (n = 7), severe (n = 7), catastrophic (n = 5)	40 (18/22)	37.90 ± 8.97 y	Blood	CBC	NLR ↔ tinnitus severity	NLR
Bayram et al., 2015 [34]	51 (26/25)	43.47 ± 13.74 y	34/17	27.02 ± 34.99 m	TRQ 28.22 ± 19.28. THQ 845.18 ± 565.99	42 (24/18)	45.19 ± 9.71	Blood	CBC		lymphocytes, MPV, neutrophils, NLR, platelet count, PLR,
Çeçen et al., 2021 [35]	74 (46/28)	52.4 ± 13.4	?	≥0.5 m	THI: 51.5 ± 21.1	65 (33/30)	50.6 ± 14.8	Blood	CBC	MPV ↑, MCV ↑	NLR, PLR, RCDW
Chrbolka et al., 2020 [36]	40	Mean 50.86 (incl. controls)	?	NR	?	40	Mean 50.86 (incl. patients)	Blood	NM		CRP
Demir, 2021 [37]	HFHL-TN 55 (37/18)	42.20 ± 11.62 y	?	0–3 m	?	57 (34/23)	37.42 ± 7.49 y	Blood	CBC	NLR ↑, PLR ↑, MPV ↑	
	AFHL-TN 53 (28/25)	43.30 ± 12.67 y	?	0–3 m	?					NLR ↑, PLR ↑, MPV ↑	
	NH-TN 51 (25/26)	41.23 ± 13.09 y	?	0–3 m	?						MPV, NLR, PLR
Düzenli et al., 2018 [38]	58 (29/29)	38.8 ± 9.41 y	0/58	≥6 m	?	58 (29/29)	38.8 ± 9.41 y	Blood	CBC	NLR ↓, PDW ↑	MPV, Platelet count, RCDW.
Gunes et al., 2019 [39]	52 (25/27)	41.62 ± 4.76 y	?	≥12 m	?			Blood	CBC	MPV (no hearing loss right ear) 7.52 ± 1.93, MPV (hearing loss right ear) 7.78 ± 1.29, MPV (no hearing loss left ear) 7.52 ± 1.73, MPV (hearing loss left ear) 7.84 ± 1.02, NLR (no hearing loss right ear) 1.99 ± 0.61, NLR (hearing loss right ear) 1.64 ± 0.71, NLR (no hearing loss left ear) 1.89 ± 0.86, NLR (hearing loss left ear) 1.53 ± 0.47	
Haider et al., 2020 [31]	92 (46/46)	Median 63 y, p25 59.8 y, p75 68.3 y (incl. controls)	40/44, 8 ?	93.6 ± 103.2 m	THI: slight (n = 17), mild (n = 38), moderate (n = 22), severe (n = 14), catastrophic (n = 1)	22 (8/14)	Median 63 y, p25 59.8 y, p75 68.3 y (incl. tinnitus patients)	Serum	Bead based multiplex assay	IL-10 ↓, IL-10 ↔ tinnitus duration	IFN-γ, IL-1α, IL-1β, IL-2, IL-6, TGF- β, TNF- α
Kemal et al., 2016 [28]	86 (46/40)	33.58 ± 11.75 y	?	?	?	84 (36/48)	32.24 ± 10.18 y	Blood	CBC	MPV ↑	RCDW, WBC
Li et al., 2019 [40]	100 (59/41)	41.62 ± 4.76 y	55/45	2.52 ± 0.72 y, range 0.3–10 y	THI: 58.78 ± 11.36 (control n = 50) and 57.26 ± 10.48 (intervention n = 50)			Serum	ELISA	Before treatment: IL-2 (control) 12.4 ± 2.65, IL-2 (intervention): 13.01 ± 3.04	
Ozbay et al., 2015 [41]	107 (35/72)	38.7 ± 12.7 y	?	≥0.5 m	THI ≥ grade 3	107 (24/83)	35.8 ± 13.9 y	Blood	CBC	NLR ↑	Lymphocytes, MPV, neutrophils, platelet count, WBC
Sarıkaya et al., 2016 [27]	101 (47/54)	40.87 ± 14.13 y	?	Mean 33.86 m, range 3–240 m	?	54 (18/36)	42.35 ± 8.94 y	Blood	CBC	MPV ↑	Platelet count
Savastano et al., 2006 [30]	36 (19/17)	41.0y, range 20–65 y	?	?	?	20 (11/9)	Mean 43.9 y, range 20–65 y	Blood	?	Circulating immune complexes ↑	CRP, Erythrocyte sedimentation rate
Savastano et al., 2007 [29]	85 (46/39)	48.36 ± 12.57 y	?	≥6m	?			Blood	?	CD3 70.70 ± 7.27 (nv 68–82), CD4 44.17 ± 7.96 (nv 36–52), CD4/CD3 0.85 ± 0.34 (nv 0.62–1.42), CD3 + CD8 23.27 ± 8.80 (nv 20–34), CD19 12.6 ± 4.20 (nv 5–16), CD16 + CD56NK 15.88 ± 7.25 (nv 1.5–15), CD3 + CD16 + CD56 4.23 ± 4.12 (nv 1.5–2.1).	
Szczepek et al., 2014 [26]	30 (16/14)	47y (range 18–67 y)	6/24	Mean 60 m, range 9–336 m	TQ: 35.4 ± 17.1			Blood	ELISA	IL-1β 4.00 ± 0.43, IL-6 0.38 ± 0.006, TNF-α 1.58 ± 1.12	
Ulusoy et al. (2018) [42]	64 (33/31)	Median 45, range 18–65 y	?	?	?	64 (38/26)	Median 41	Blood	CBC	MPV ↑, PDW ↑	NLR, platelet count, PLR, WBC count
Weber et al., 2002 [32]	26 (16/10)	32.2 ± 9.7 y	?	?	TQ: 30.50 ± 14.38	13 (8/5)	32.0 ± 6.7 y	Serum	ELISA	IL-6 ↑	IL-10, TNF- α
Yildiz et al., 2020 [43]	287 (119/168)	44.89 ± 10.96	?	?	?	257 (130/127)	38.37 ± 10.65	Blood	CBC	NLR ↑, % of high MPV within the group ↑	
Yüksel and Karataş, 2016 [44]	100 (57/43)	50.95 ± 14.6 y	(66/34)	35.90 ± 44.49 m	THI 41.62 ± 14.65. Slight (n = 2), mild (n = 37), moderate (n = 43), severe (n = 18), catastrophic (n = 0)	100 (39/61)	44.39 ± 8.9 y	Blood	CBC	MPV ↓, PDW ↑, Platelet count ↑, CRP ↑, sedimentation ↑	

Abbreviations: AFHL-TN, all frequency hearing loss tinnitus; Bi, bilateral; CBC, complete blood count; CRP, C-reactive protein; ELISA, enzyme-linked immunosorbent assay; F, female; Hb, hemoglobin; HFHL-TN, high frequency hearing loss tinnitus; IFN, interferon; IL, interleukin; M, male; m, months; MCV, mean corpuscular volume; MPV, mean platelet volume; NH-TN, normal hearing tinnitus; NLR, neutrophil-to-lymphocyte ratio; NM, not mentioned; NR, assessed but not reported; nv, normal value; PDW, platelet distribution width; PLR, platelet-to-lymphocyte ratio; RCDW, Red cell distribution width; TGF, transforming growth factor; THI, tinnitus handicap inventory; THQ, Tinnitus handicap questionnaire; TNF, tumor necrosis factor; TQ, tinnitus questionnaire; TRQ, tinnitus reaction questionnaire; Uni, unilateral; WBC, white blood cell count; y, years.

### 3.3. Meta-Analysis Complete Blood Count (CBC) Markers

**Figure 2:**
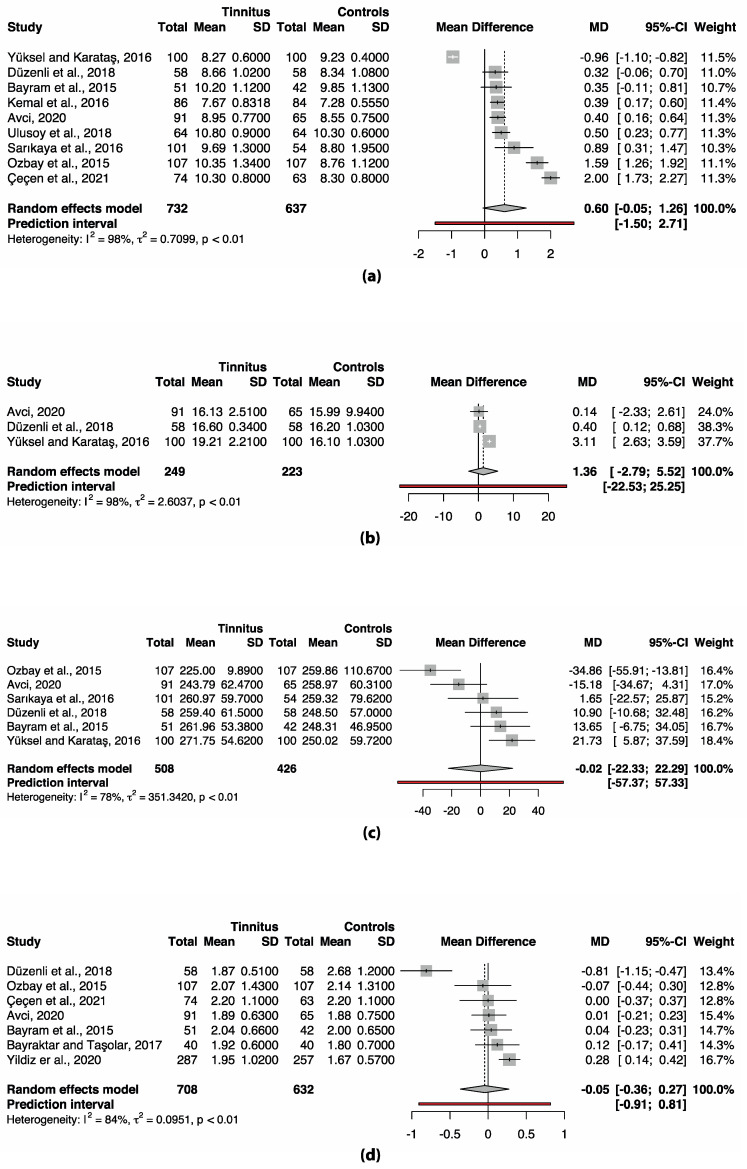
Forest plots of (**a**) MPV, (**b**) PDW, (**c**) platelet count and (**d**) NLR. No significant differences are present in any of the markers. Abbreviations: CI, confidence interval; MD, mean difference; SD, standard deviation.

Only the data for the human complete blood count markers could be meta-analyzed due to the heterogeneity of the data. Included markers were MPV, PDW, platelet count and NLR. The study of Ulusoy et al. (2018) could not be included in the meta-analysis of PDW, platelet count and NLR, since this data was not normally distributed [42]. None of the markers differed significantly between participants with and without tinnitus (Figure 2). However, the meta-analysis of all markers showed considerable heterogeneity (*I*^2^ 78–98%, Figure 2). This warrants further analysis of the between-study heterogeneity. Since there are multiple options to analyze heterogeneity, and the choice for one option over another is arbitrary, a multiverse analysis was performed. The multiverse analysis of heterogeneity assessment is depicted in Table 3. When the considerable between-study heterogeneity in MPV is assessed, both methods result in a significant difference by removing one to three studies from the model. Moreover, in PDW this only results in a significant difference after influence analysis and removing the influential study. In platelet count and NLR, the lack of differences between the tinnitus and control group remains in all applied methods.

**Table 3:** Multiverse analysis of between-study heterogeneity assessment. MPV differences became significant after both outlier removal and influential study removal. PDW difference became significant after removal of an influential study. Significant values are marked with an asterisk (*).

Analysis	MD	95% CI	*p*	95% PI	*I* ^2^	95% CI	Removed Study
*MPV*							
Main analysis	0.602	−0.052–1.258	0.067	−1.450–2.705	98.4%	97.9–98.8%	
Basic outlier removal	0.426	0.297–0.556	<0.001 *	0.286–0.567	0%	0–74.6%	Çeçen et al. (2021) [35] Ozbay et al. (2015) [41] Yüksel and Karataş (2016) [44]
Influence analysis	0.807	0.261–1.353	0.010 *	−0.846–2.459	94.9%	92.1–96.8%	Yüksel and Karataş (2016) [44]
*PDW*							
Main analysis	1.360	−2.795–5.520	0.294	−22.534–25.255	97.8%	95.9–98.9%	
Basic outlier removal	1.360	−2.795–5.520	0.294	−22.534–25.255	97.8%	95.9–98.9%	No outliers detected
Influence analysis	0.397	0.028–0.765	0.047 *	-	0%	-	Yüksel and Karataş (2016) [44]
*Platelet count*							
Main analysis	−0.019	−22.329–22.291	0.998	−57.369–57.331	77.9%	51.1–90.0%	
Basic outlier removal	−0.019	−22.329–22.291	0.998	−57.369–57.331	77.9%	51.1–90.0%	No outliers detected
Influence analysis	7.155	−10.966–25.275	0.335	−33.967–48.276	55.3	0.0–83.5%	Ozbay et al. (2015) [41]
*NLR*							
Main analysis	−0.046	−0.365–0.273	0.736	−0.907–0.815	83.6%	67.7–91.7%	
Basic outlier removal	0.103	−0.042–0.248	0.127	−0.219–0.425	34.6%	0.0–73.8%	Düzenli et al. (2018) [38]
Influence analysis	0.103	−0.042–0.248	0.127	−0.219–0.425	34.6%	0.0–73.8%	Düzenli et al. (2018) [38]

Abbreviations: CI, confidence interval; MD, mean difference; MPV, mean platelet volume; NLR, neutrophil-to-lymphocyte ratio; PDW, platelet distribution width; PI, prediction interval.

## 4. Discussion

**Figure 3:**
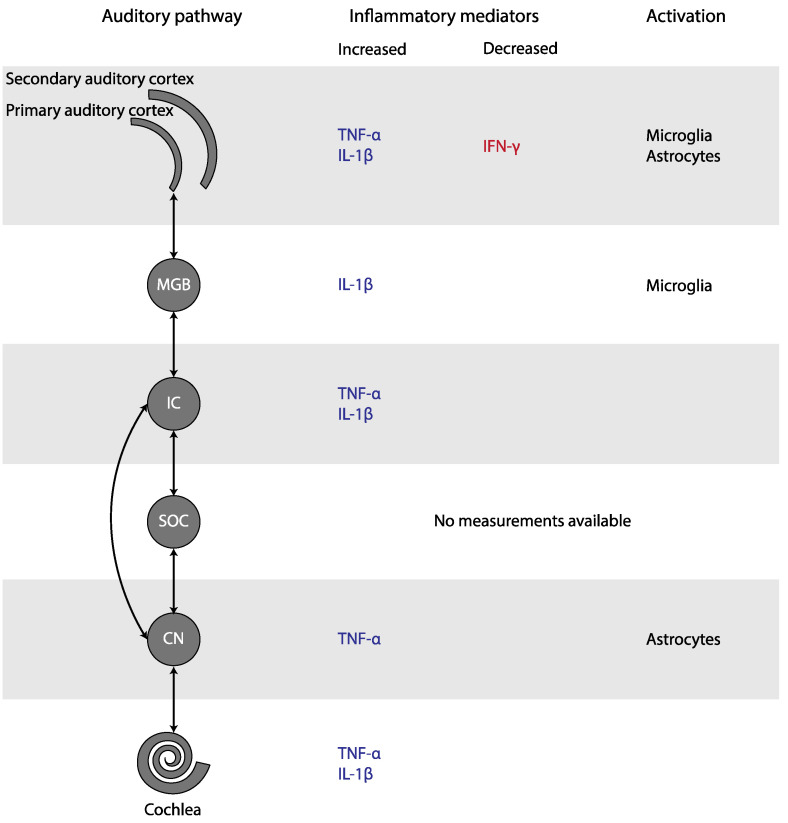
Changes in inflammatory mediators and glial activation in tinnitus, depicted in the simplified auditory pathway. Abbreviations: CN, cochlear nucleus; IC, inferior colliculus; IFN, interferon; IL, Interleukin; MGB, medial geniculate body; SOC, superior olivary complex; TNF, Tumor Necrosis Factor.

In this systematic review, we studied the presence of inflammation in subjective tinnitus. Experimental studies found an increase of TNF-α in the cochlea, the CN, the IC, and the AC, as well as an increase of IL-1β in the cochlea, the IC, the MGB, and the AC of animals with tinnitus (Figure 3). In addition, microglial markers were increased in the MGB and AC, but decreased in the CN. Astrocytic markers were elevated in the CN and AC. Only human CBC measurements could be meta-analyzed. MPV and PDW in blood samples were increased only if outliers/influential studies were removed. Together, these results indicate that inflammation may play a role in tinnitus.

### 4.1. Cytokine Involvement in Tinnitus

#### 4.1.1. TNF-α in Tinnitus

TNF-α is a potent pro-inflammatory molecule produced by brain-resident astrocytes, microglia, and neurons [45]. It appears to play a critical role in the pathogenesis of experimental tinnitus. TNF-α was consistently increased after tinnitus induction and infusion of TNF-α in the AC of healthy mice resulted in tinnitus [16,17,18,19,46]. Moreover, genetic deletion and pharmacological blockade of TNF-α prevented the occurrence of tinnitus and noise-induced microglial activation. After infusion of TNF-α, these animals developed tinnitus as well. Additionally, pharmacological depletion of microglia before noise exposure prevented a TNF-α increase and noise-induced tinnitus [16]. These results suggest that microglia are (for a large part) responsible for increased TNF-α expression, and that microglial activation and TNF-α depend on each other in tinnitus induction [16]. Once TNF-α is present, it is able to activate parenchymal microglia, forming a self-stimulating loop [47].

In contrast with the experimental studies, there was no increase in TNF-α present in humans with tinnitus. This discrepancy may be caused by the timing of measurements relative to the start of tinnitus. In animals, the increases in TNF-α were detected almost directly after tinnitus induction in most cases, while in humans, cytokine levels were measured in subjects with chronic tinnitus, i.e., the measurements took place much longer after the start of tinnitus. TNF-α levels may have been elevated initially and reduced to normal levels in the chronic state. Additionally, in animal models, cytokine levels were measured in brain tissue, whereas human cytokine levels were measured in blood. Last, damage because of the tinnitus induction methods in animals itself could be responsible for an increase in TNF-α, independent of the occurrence of tinnitus. However, animals that were treated the same but did not develop tinnitus also did not show an increase in TNF-α. Taken altogether, it is probable that noise exposure and salicylate administration cause an increase in TNF-α, which leads to acute tinnitus. In contrast, in chronic tinnitus TNF-α may not play a role anymore.

#### 4.1.2. IL-1β in Tinnitus

IL-1β (gene) expression was consistently increased in experimental tinnitus. Similar to TNF-α, IL-1β was not increased in humans, although it was only evaluated in one study [31]. IL-1β is a potent pro-inflammatory cytokine that stimulates microglia and astrocytes, and stimulates the expression of inflammatory mediators, including itself [48]. It can be produced by microglia, astrocytes, endothelial cells, infiltrating leukocytes, neurons, and oligodendrocytes [49]. Since IL-1β is hardly detectable in healthy brains [48,50], the elevation in its (gene) expression levels in rats with tinnitus suggests that IL-1β is present in tinnitus. The positive association between tinnitus scores and IL-1β levels in mice seems to confirm this [20]. However, it is not clear whether the increased (gene) expression of IL-1β (partly) caused tinnitus or vice versa. For the difference between IL-1β expression between human and animal studies, the same reasons as mentioned for TNF-α could apply.

### 4.2. Neuroglial Involvement in Tinnitus

Microglial activation is characterized by the increased expression of specific markers, such as Iba-1, and morphological changes from the ramified shape to a non-ramified shape. The number of Iba-1 positive microglia and their activation state were increased in the MGB and in the primary AC, but decreased in the CN. After activation, microglia produce proinflammatory cytokines such as TNF-α. These cytokines further activate the microglia, forming a self-stimulating loop [16]. The central role of microglia is further supported by the observation that microglial depletion prevented an increase in TNF-α expression and noise-induced tinnitus [16]. Similar to microglial activation, astrocytic activation is characterized by increased expression of specific markers, such as GFAP, and deramification of their processes [51]. The number of GFAP-positive astrocytes and their activation state were increased in the ventral CN and primary AC, but not in the MGB. Astrocytes are important in regulating levels of neurotransmitter and ion concentrations, controlling synapse formation and function, and repairing the nervous system [52,53]. In response to neuronal degeneration, they promote synaptic regrowth and axonal sprouting [22,54]. This may lead to enhanced synaptic activity. Moreover, astrocytes can directly excite neighboring neurons through a calcium-dependent glutamate release and promote neural synchrony mediated by extra-synaptic excitatory receptors [55,56]. Furthermore, they can produce the proinflammatory cytokines implicated in tinnitus [57]. Thus, both microglia and astrocytes may play an important role in tinnitus.

### 4.3. Platelet Involvement in Tinnitus

In humans with tinnitus, MPV was increased in six out of ten studies, but this was only significant when outliers or influential cases were removed. PDW was increased in three out of four studies that examined this marker, but like MPV this was only significant when an influential study was removed. MPV indicates the size of platelets, whereas PDW reflects variation of platelet size distribution. Since platelets change their shape when activated, increased MPV and PDW can both be used as a sign of activated platelets. In line with these results, Chrbolka et al. (2020) showed that platelet activity was increased in patients with tinnitus [36]. Platelet count and NLR seem to remain unchanged.

Platelets may be involved in the development of tinnitus in several ways. Platelets release granule-stored cytokines such as IL-6, IL-8 and TNF-α within seconds to minutes after activation. In addition, they produce various chemokines and cytokines, such as IL-1β, within hours after activation [58,59]. These cytokines, and thus platelets, are potent inducers of the acute phase response [60]. Therefore, platelets are an important source of cytokines in the inflammatory response. On the other hand, increased platelet volumes may be an indicator of a prothrombic condition or even cause thrombosis, e.g., in the internal auditory artery leading to hypoperfusion of the cochlea, impairing its function and contributing to the development of tinnitus [28,38,42]. Finally, platelets are involved in glutamate uptake. Reduced systemic glutamate uptake by platelets, as well as glutamate excitotoxicity, has been demonstrated in various neurodegenerative disorders, such as Alzheimer’s disease, Parkinson’s disease, and amyotrophic lateral sclerosis [61]. Although speculative, reduced systemic glutamate uptake by platelets could play a role in tinnitus as well, given the elevated glutamate concentration in tinnitus [62].

### 4.4. Inflammation in the Pathophysiology of Tinnitus

The question remains how inflammation would be involved in the pathophysiology of tinnitus. Tinnitus has been associated with an increase in excitatory and a decrease in inhibitory neurotransmission [62]. Inflammation, on the other hand, may lead to alterations in synaptic transmission and synaptic organization.

**Figure 4:**
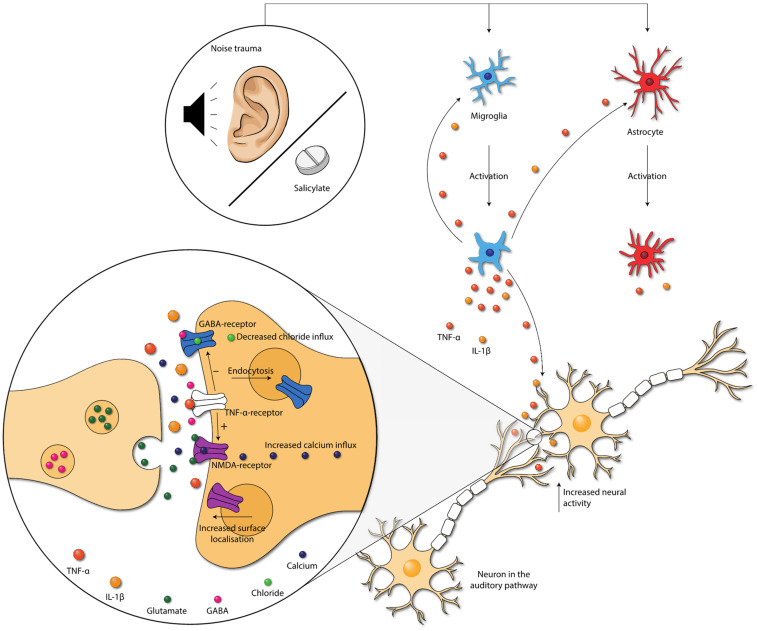
Damage caused by noise exposure and salicylate administration leads to the activation of neuroglia. Neuroglia produce cytokines that further activate the neuroglia. Moreover, cytokines influence neurotransmission within the auditory pathway. TNF-α increases the NMDA-receptor and decreases the GABA_A_-receptor activity, via the TNF-α-receptor. Moreover, NMDA receptor surface localization increases, and GABA-receptor surface localization decreases. These changes lead to increased neural activity in the auditory pathway. Abbreviations: GABA, gamma-aminobutyric acid; IL, interleukin; NMDA, N-methyl-D-aspartate; TNF, tumor necrosis factor.

Salicylate-induced tinnitus is associated with the increased activity of the NMDA (N-methyl-D-aspartate) receptor, one of the excitatory glutamate receptors [63] (Figure 4). Some animal studies included in this review also studied the (gene) expression of NR2A and NR2B, two NMDA receptor subunits. Both NR2A and NR2B (gene) expression were consistently elevated in the IC, the AC, and the cochlea in animals with tinnitus after salicylate injection. Moreover, a positive association between NR2B and tinnitus scores was present [17,18,19,20,23,46]. When tinnitus was diminished, (gene) expression of both NR2A and NR2B returned to normal as well. Thus, in animals with salicylate-induced tinnitus, NMDA (gene) expression is increased. Interestingly, this increase was consistently coexistent with increases in TNF-α and IL-1β in animals with tinnitus throughout the entire auditory pathway [17,18,19,46]. The specific effect of TNF-α on the NMDA receptor is still a matter of debate as both an increase and decrease of NMDA receptor currents have been demonstrated [64,65,66,67]. However, most studies show an increase. Additionally, TNF-α enhances surface localization of NMDA receptor subunits [67]. Last, Wang et al. (2019) showed that tinnitus was associated with TNF-α-dependent increased excitatory and decreased inhibitory synaptic currents [16]. Besides TNF-α, IL-1β has also been shown to enhance NMDA-induced currents [66,68]. In sum, the increased expression of TNF-α, and possibly also IL-1β, leads to increased NMDA receptor-dependent calcium influx and enhanced post-synaptic currents. This leads to increased neural activity.

The gamma-aminobutyric acid (GABA)-receptor is one of the major inhibitory neurotransmitter receptors. A potent tinnitus inducer, sodium salicylate, has been shown to suppress GABA_(A)_-induced currents, and tinnitus was completely eliminated after oral administration of the GABA agonist Vigabatrin, which suggests the involvement of decreased inhibitory neurotransmission in tinnitus [69,70,71]. Interestingly, inhibitory neurotransmission is also impacted by cytokines. TNF-α expression influences inhibitory GABA_A_ (gamma-aminobutyric acid) receptors by causing an endocytosis of GABA_A_ receptors, resulting in fewer surface GABA_A_ receptors and a decrease in inhibitory synaptic strength [72]. Additionally, IL-1β has been shown to suppress GABA-induced currents [66]. So, besides increasing excitatory neurotransmission, TNF-α and IL-1β presumably also suppress inhibitory neurotransmission in tinnitus.

Vice versa, the abnormal release and/or uptake of neurotransmitters can also result in inflammation [57]. Intraperitoneal injections of memantine, a non-competitive NMDA receptor antagonist, for seven consecutive days was successful in decreasing NR2B expression and attenuating tinnitus. It also decreased expression of TNF-α [46]. Therefore, it seems probable that salicylate and noise exposure cause both an imbalance in neurotransmission and neuroinflammation, leading to tinnitus. This could lead to a vicious circle in which inflammation and neurotransmitter imbalance reinforce each other, exacerbating tinnitus.

Changes in the activity of receptors can lead to synaptic plasticity, which is the ability of synapses to strengthen or weaken over time [73]. Therefore, the cytokine-induced changes in excitatory and inhibitory neurotransmission could influence neural plasticity associated with tinnitus [74]. Moreover, it has been stated that the proinflammatory cytokines as TNF-α and IL-1β itself may also directly alter long-term synaptic plasticity [66,75,76,77]. Synaptic plasticity may occur throughout the whole auditory pathway, both ascending and descending. The detailed, specific mechanisms and types of neuroplasticity in tinnitus are beyond the scope of this review. Therefore, we refer to the reviews of Roberts (2018) and Wu et al. (2016) on this topic [78,79]. Concluding, synaptic plasticity caused by inflammation could explain the long-term effects after the induction of tinnitus.

Various studies have reported that psychological stress increases the bothersomeness and loudness of tinnitus [80]. Baigi et al. (2011) even suggested that stress is a key factor in modulating the severity of tinnitus [81]. Acute stress stimulates the inflammatory response by increasing the amount of circulating IL-6, IL-1β, TNF-α and IL-10 [82]. On the other hand, chronic stress is also associated with increases in the amount of circulating cytokines, such as IL-6 and TNF-α [82]. Therefore, it is not implausible that the effect of stress on the severity of tinnitus is effectuated via inflammatory pathways.

In conclusion, damage caused by noise exposure and salicylate administration leads to inflammation. In turn, proinflammatory cytokines seem to influence synaptic transmission by either increasing excitatory synaptic transmission and/or decreasing inhibitory synaptic transmission throughout the whole auditory tract. Neuroplasticity occurs because of the altered activity of these receptors. This could explain the long-term effects of tinnitus.

### 4.5. Challenges in the Interpretation of Results on Inflammation in Tinnitus

#### 4.5.1. Translation from Animal to Human

When both animal studies and human studies are compared, translatability may become an issue. Whereas the included animal studies studied the effects of tinnitus in the acute phase, the human studies investigated effects of the chronic phase. Moreover, in most animal studies, tinnitus was induced by salicylate. However, in humans, most cases of chronic tinnitus are caused by noise exposure or are related to age-related hearing loss [2]. For salicylate to cause tinnitus in humans, high doses are required [83]. Even though these are valid concerns, it does not mean that animal studies are not of value for unraveling the pathophysiology of tinnitus in humans. A recent study showed that, although different methods are employed to study the activity and connectivity of brain areas, there is consistency within the results between these approaches in human and animal studies [84]. Therefore, the inflammatory effects shown in animal models for tinnitus may very well play a role in humans as well.

#### 4.5.2. Contribution of Hearing Loss

Another difficulty in the interpretation of results on inflammation in tinnitus is the potential contribution of hearing loss. As stated before, the pathophysiology of tinnitus and that of acquired hearing loss are closely related to such an extent that they often co-exist. Moreover, inflammation has been implicated in the pathophysiology of hearing loss as well [5,6]. Therefore, it is a challenge to disentangle both pathophysiological mechanisms.

Animal studies included in this review showed that both salicylate administration and noise trauma not only induce tinnitus, but also affect hearing thresholds [16,22,24,46,85]. In the study of Wang et al. (2019), TNF-α knockout mice had a similar auditory brainstem response as wild type mice after noise exposure, but the TNF-α knockout mice did not develop tinnitus, whereas wild type mice did [16]. This proves that TNF-α is essential for the development of tinnitus, but not for hearing loss. Therefore, elevated levels of TNF-α can (also) be attributed to the presence of tinnitus in animals.

In many human studies, hearing thresholds were not reported, and patients with hearing loss were not excluded. Only Kemal et al. (2016), Yildiz et al. (2020), Bayraktar and Taşolar (2017) and Chrbolka et al. (2020) excluded patients with a pure tone average over 20dB, 25dB and 40dB, respectively [25,28,36,43]. Moreover, Ceçen et al. (2021), Ozbay et al. (2015) and Savastano et al. (2006) excluded patients with (moderate or severe) hearing loss, but did not provide cut-off values [30,35,41]. Studies comparing human cytokine levels between patients with and without tinnitus did also not account for hearing loss in their analysis [31,32]. Blood cytokine levels in humans with tinnitus may not be altered (except for IL-6 in one study), but a potential effect of tinnitus on the presence of cytokines may also be masked by the simultaneous presence of hearing loss. To conclude, patients included in the human studies are very heterogenic, and it cannot be ruled out that the presence of hearing loss affects the results.

### 4.6. Potential Treatment Options

Given the role of TNF-α, IL-1β and microglia in the tinnitus pathophysiology, these mediators could potentially be a target for treatment. Whereas inflammation seems to play a role in the acute phase of tinnitus, it is less clear in the chronic phase. Therefore, inflammatory targets for treatment may be mainly beneficial in acute tinnitus.

#### 4.6.1. Treatments Targeting Cytokines

Commonly used TNF-α inhibitors include adalimumab, certolizumab pegol, etanercept, golimumab, and infliximab. With regard to tinnitus, only etanercept has been studied. Hwang et al. (2017) showed that intraperitonially injected Etanercept significantly reduced behavioral evidence of tinnitus in mice [24]. Alongside gene expression of TNFR1, TNFR2 and NR2B was reduced. Etanercept has been shown to be effective in patients with Alzheimer’s disease, in which it was suggested that it reversed neuronal excitability associated with TNF-α exposure by acting as a decoy receptor [86]. Currently, the first clinical trial is planned to study the effect of etanercept in humans with blast-induced tinnitus (ClinicalTrials.gov, identifier: NCT04066348).

Another drug targeting TNF-α is dithiothalidomide (dTT). DTT is an immunosuppressive used to treat multiple myeloma and erythema nodosum leprosum, among other afflictions. In mice, dTT injections for five days within an hour after noise exposure prevented the increases in TNF-α, IL-1β, IL-18, NLRP3 and microglial morphological changes in the primary AC. Moreover, the mice showed no behavioral evidence of tinnitus [16]. In rats, dTT did not prevent the onset of tinnitus when administered directly after noise exposure, but it did alleviate subsequent tinnitus [16]. These studies indicate that TNF-α-inhibitors may be helpful in treating tinnitus or preventing its onset.

Anakinra is a competitive IL-1-type-1-receptor antagonist which inhibits the binding of IL-1α and IL-1β. It is mainly used to treat rheumatoid arthritis. Canakinumab is a human monoclonal antibody targeting IL-1β which is used in the treatment of systemic juvenile idiopathic arthritis and active Still’s disease. However, no study on the effect of IL-1β antagonists on tinnitus has been performed yet, either in animals or in humans.

#### 4.6.2. Treatments Targeting Microglia

Prevention of microglial activation may be a potential treatment strategy. Intrathecal administration of Minocycline, a broad-spectrum tetracycline antibiotic, inhibits the expression of pro-inflammatory cytokines by microglia, leading to reduced attenuated mRNA expression and decreased cerebrospinal fluid levels of IL-1β and TNF-α [87]. However, it has not been studied with regard to tinnitus.

### 4.7. Strengths and Limitations 

To our knowledge, this is the first systematic review and meta-analysis to evaluate the presence of inflammation in tinnitus. Several limitations of this review should also be considered. First, we could only include one article that studied noise-induced tinnitus in animals. This is partly due to our strict inclusion criteria. To prevent a selection bias, we only included animal studies that performed a tinnitus evaluation, even though they used (for a large part) the same noise exposure paradigm as studies that did not. Similar studies without the word tinnitus in their article would not come up in the search results. Moreover, due to the large heterogeneity of study designs, only a meta-analysis of the complete blood count markers was possible. Additionally, inflammatory mediators have only been studied in the brain tissues of animals, but not yet in the brain tissues of tinnitus patients.

## 5. Conclusions

Accumulating evidence suggests that inflammation plays a role in the pathogenesis of subjective tinnitus. Noise exposure and salicylate administration both lead to inflammation throughout the whole auditory pathway. In particular, TNF-α, IL-1β, glia and activated platelets are associated with acute tinnitus. TNF-α and IL-1β influence NMDA and GABA_A_ receptors, leading to an increased excitatory and decreased inhibitory neurotransmission. These changes can lead to neuroplasticity and thus chronic tinnitus. Whether inflammatory mediators still play a role in chronic tinnitus remains to be elucidated. Nevertheless, drugs targeting the involved inflammatory mediators could be a potential effective treatment for (acute) tinnitus.

## Data Availability

The data presented in the study were obtained from the included trials and are openly available. The code that was used for the processing of the data can be provided upon request.
